# Casticin Impacts Key Signaling Pathways in Colorectal Cancer Cells Leading to Cell Death with Therapeutic Implications

**DOI:** 10.3390/genes13050815

**Published:** 2022-05-03

**Authors:** Michael Kowalski, Ashley Assa, Ketki Patil, Courtney Terrell, Nathan Holliday, S. Balakrishna Pai

**Affiliations:** Wallace H. Coulter Department of Biomedical Engineering, Georgia Institute of Technology, Emory University, Atlanta, GA 30332, USA; mikekowalski98@gmail.com (M.K.); ashlz11@yahoo.com (A.A.); kpatil7@mail.gatech.edu (K.P.); terrell.courtney.ct@gmail.com (C.T.); nholliday314@gmail.com (N.H.)

**Keywords:** casticin, DLD-1, HCT116, Caco-2, colorectal cancer, Bcl-2, apoptosis, colony forming efficiency, cell cycle

## Abstract

Colorectal cancer is the third most frequently encountered cancer worldwide. While current chemotherapeutics help to manage the disease to some extent, they have eluded achieving complete remission and are limited by their severe side effects. This warrants exploration of novel agents that are efficacious with anticipation of minimal adverse effects. In the current study, casticin, a tetramethoxyflavone, was tested for its ability to inhibit the viability of three human colorectal cancer cells: adenocarcinoma (DLD-1, Caco-2 cell lines) and human colorectal carcinoma cells (HCT116 cell line). Casticin showed potent inhibition of viability of DLD-1 and HCT116 cells. Clonogenic assay performed in DLD-1 cells revealed that casticin impeded the colony-forming efficiency of the cells, suggesting its impact on the proliferation of these cells. Further, a sustained effect of the inhibitory action upon withdrawal of the treatment was observed. Elucidation of the mechanism of action revealed that casticin impacted the extrinsic programmed cell death pathway, leading to an increase in apoptotic cells. Further, Bcl-2, the key moiety of cell survival, was affected. Notably, a significant number of cells were arrested in the G2/M phase of the cell cycle in DLD-1 cells. Due to the multifaceted action of casticin, we envision that treatment with casticin could provide an efficacious treatment option for colorectal adenocarcinomas with minimal side effects.

## 1. Introduction

Cancer is the second leading cause of death globally, affecting an estimated 2.6 million people each year. Specifically, colorectal cancer accounts for nearly 1.8 million new cases and is the third leading cause of cancer related death at 862,000 every year, which has increased dramatically from 2012 when the incidence rate was over 1 million cases with 694,000 deaths worldwide [1]. Diagnosing colorectal cancer at an early stage is considered beneficial, because neoadjuvant chemotherapy can be administered [2], but the relapse of cancer observed in many patients is of great concern. Since 5-FU is one of the choices of chemotherapeutic drugs administered to treat aggressive type of colorectal cancers [3], resistance to it poses a major problem [4]. Therefore, to treat colorectal cancers efficaciously, better treatment options are needed. Furthermore, discovering novel entities with potent action to combat this malady are essential. Natural compounds are being researched for potential cancer treatments and have shown promise as cancer therapeutics. In fact, 74% of the most important lifesaving drugs consist of plant-derived active ingredients; moreover, natural compounds are being used to treat 87% of all categorized human diseases [5]. Through many biological, medicinal, and pharmacological uses, it has become clear that natural compounds have anticancer and antibacterial properties [6,7]. An example of an anticancer property in natural compounds, such as flavonoids, is its ability to induce apoptosis and cell cycle arrest in cancer cells, while not causing cytotoxic effects in normal healthy cells [8]. 

Casticin (a specific flavonoid-tetramethoxyflavone), and other flavonoids have been reported to induce either apoptosis or cell cycle arrest in ovarian, breast, gallbladder, liver and other colorectal cancer cells [9,10,11,12,13,14,15]; however, no research has been done on the effect of casticin on DLD-1 cells. The present study is motivated by this lack of information. This study was undertaken to determine the efficacy of casticin on colorectal cancer cell lines and elucidate its mechanism of action with the aim of achieving an efficacious treatment option for colorectal cancers with potentially fewer side effects.

## 2. Materials and Methods

### 2.1. Cell Lines and Materials

The colorectal adenocarcinoma (DLD-1), colorectal carcinoma (HCT116), and colorectal adenocarcinoma (Caco-2) cell lines were obtained from the American Type Culture Collection (ATCC). For DLD-1 cells, the growth medium was prepared by supplementing RPMI with 10% FBS, 1% Penicillin/Streptomycin, and 2 mM L-Glutamine. The HCT116 cell line was maintained in McCoy’s 5A medium supplemented with 10% FBS, 1% Penicillin/Streptomycin, and 2 mM L-Glutamine. Caco-2 cells were grown in EMEM with 20% FBS and 1% Penicillin/Streptomycin antibiotic solution. Cell cultures were maintained at 37 °C in an atmosphere of 5% CO_2_ and 95% air. Casticin was purchased from MedChem Express and dissolved in DMSO to prepare a stock solution of 20 mM.

### 2.2. Cytotoxicity Assessment of Casticin on Colorectal Cancer Cell Lines DLD-1, HCT116 and Caco-2

Cytotoxicity assays were performed as described earlier [16,17]. Cells were plated in triplicate for control and casticin treatments in 96-well plates at 5000 cells per well. After the cells adhered during the 24 h of incubation, the medium was replaced with fresh media for the control group or media with varying concentrations of casticin for the treatment groups. The cells were incubated for 48 h, after which cell viability was assessed using Cell Counting Kit-8 (CCK-8) assay per Bimake CCK-8 protocol B34305.

### 2.3. Clonogenic Assay

DLD-1 cells were plated at 100,000 per well and were treated in triplicate for 48 h with 5 μM of casticin. After the treatment period, cells were trypsinized and counted. To determine the efficiency of the treated cells to form colonies when compared to the control cells, cells were then plated in 6-well plates at a density of 500 cells per well and incubated for 10 days in media without any compounds. After this period, media was removed and cultures were washed with PBS, fixed with ice-cold methanol, and stained with crystal violet solution for visualization of colony formation. Detailed procedure for the determination of colony-forming efficiency has been described earlier [18].

### 2.4. Muse^®^ Flow Cytometric Analysis

The flow cytometric analyses used in this study are Annexin V & Dead Cell assay, Multicaspase assay, Bcl-2 Activation Dual Detection assay, and Cell Cycle Analysis (MCH100105, MCH100109, MCH200105, and MCH100106) from EMD Millipore. Assays were performed as per protocols published earlier [19]. Cells were plated at 2 × 10^5^ cells per well in triplicate in 12-well culture plates. Control groups received the growth media, and for the treatment group, 5 μM of casticin in growth media was added. After 48 h of incubation, the cytometric analyses were performed with a Muse cell analyzer as per the manufacturer’s instructions. 

### 2.5. Statistical Analysis

To determine the statistical significance of the data, GraphPad Prism was used to perform statistical analysis. Mean values ± standard deviations were calculated, and the *p*-values are mentioned in the legend section of each figure.

## 3. Results

### 3.1. Impact of Casticin on the Viability of DLD-1, HCT116, and Caco-2 Cells

**Figure 1:**
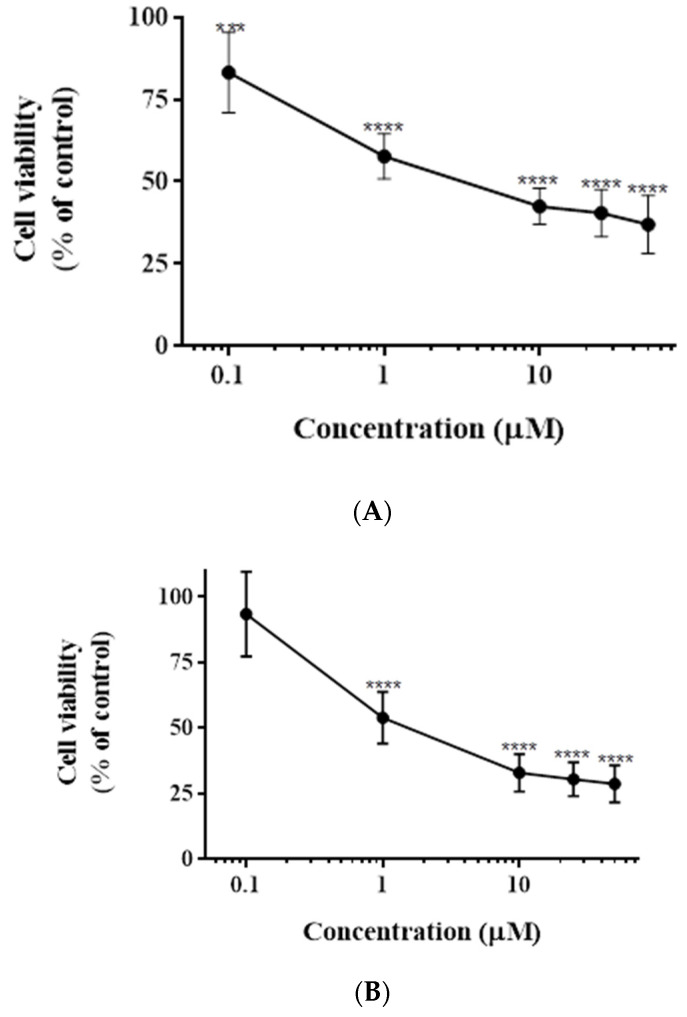

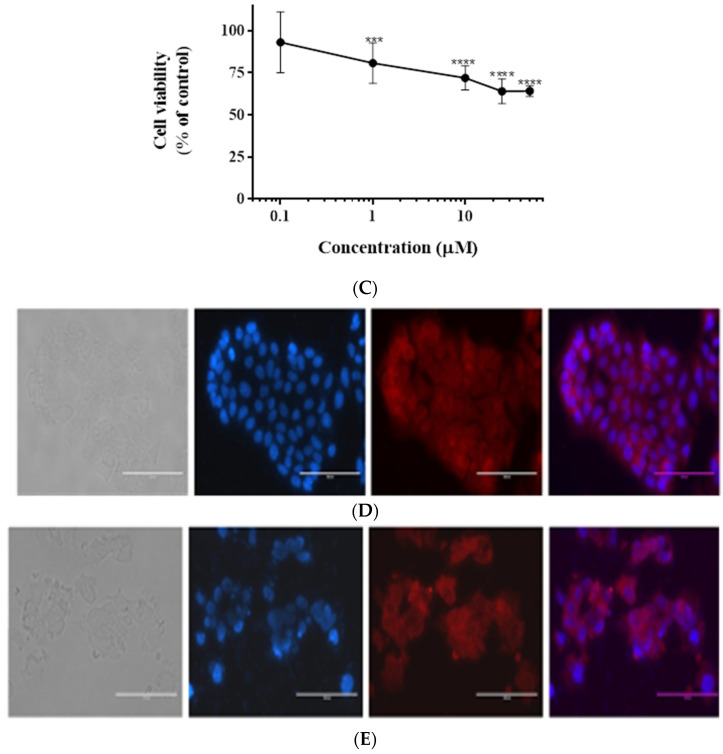
Cytotoxicity profile of casticin on various colorectal cancer cell lines. Cytotoxicity of casticin on DLD-1 (colorectal adenocarcinoma) cells was tested. Mean values ± standard deviations from 7 trials with p values of 0.0003 (***) and <0.0001 (****) when compared to control are shown in (**A**), cytotoxicity of casticin on HCT116 (colorectal carcinoma) cell line; mean values of 11 trials with a *p* value < 0.0001 (****) for concentrations beginning 1 µM onwards when compared to control (**B**), cytotoxicity of casticin on Caco-2 cell line with p values of 0.0007 (***) and <0.0001 (****) is shown in (**C**). Cytotoxicity was determined using the CCK-8 assay. Further, confocal images of the control DLD-1 sample and treated samples were taken at 40X magnification. Image of DLD-1 control cells are shown in (**D**), image of the casticin-treated sample (**E**). View from the horizontal panel depicts phase contrast images of each group followed by DAPI stain, Phalloidin stain, and a merged image of the two stains. The scale bar is 100 μm. When compared to the control sample, as seen in the images of the treated sample, the cells are less in number. One-way ANOVA with Dunnett’s multiple comparisons test with alpha set to 0.05 was employed for the analysis of (**A**–**C**) datasets.

The potential for inhibition of viability of human colorectal cancers cells DLD-1, HCT116, and Caco-2 by casticin was tested by treating the cells at varying concentrations of the compound. Cell viability was measured by CCK-8 assay. DLD-1 cells were sensitive to casticin with IC_50_ concentration of 5 μM as shown in Figure 1A, whereas HCT116 cells exhibited an IC_50_ concentration of 1.5 μM (Figure 1B). Casticin induced negligible amounts of toxicity to Caco-2 cells (Figure 1C). Further, confocal images of the DLD-1 control sample and treated samples were taken at 40 X magnification. Image of DLD-1 control cells are shown in (Figure 1D), and an image of the casticin-treated sample in (Figure 1E). As seen in the images, the treated cells are less in number.

### 3.2. Casticin Treatment Impacts Colony-Forming Efficiency of DLD-1 Cells

**Figure 2:**
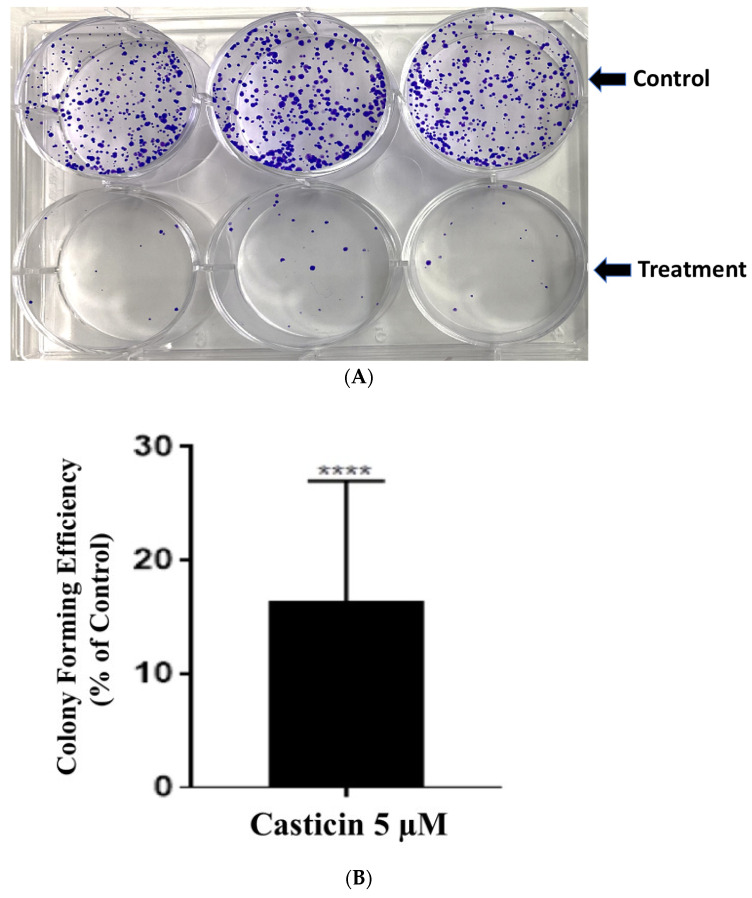
Casticin treatment impacts colorectal cancer cell proliferation. To study the effect on colony-forming ability and sustained effect of casticin on DLD-1 cells, clonogenic assays were performed. The cells were treated for 48 h in each treatment, subcultured, and allowed to proliferate for 10 days after the replacement of the treated media with normal media. (**A**) Representative image of the control cultures and cultures from casticin (5 µM) treatment for 48 h. (**B**) The colony-forming efficiency when compared to the control sample. Colonies were counted and the mean values ± the standard deviation of 6 trials are shown. Two-tailed unpaired *t*-test was performed. Colonies in casticin-treated sample decreased significantly from the control: *p*-value = 0.0001 (****).

On observing the potent inhibition of cell viability by casticin, we proceeded to study its impact on the proliferation potential of DLD-1 cells by performing colony forming efficiency assay. For this assay, the cells were treated at the IC_50_ concentration of casticin (5 μM) and incubated for 48 h and then were replated in fresh media in the absence of casticin and incubated for 10 days (as described in Section 2). In Figure 2, representative images of all treatments for DLD-1 are shown. Control sample and treatment with casticin 5 µM (Figure 2A). The data indicates that casticin continued to impact the cellular proliferation of DLD-1 cells after the initial 48 h treatment period even on withdrawal of the treatment. The colony-forming efficiency when compared to the control sample is represented in (Figure 2B). Colonies in the casticin-treated sample decreased significantly as compared to the control. A sustained response was observed even upon the withdrawal of casticin.

### 3.3. Casticin Invokes Extrinsic Programmed Cell Death Pathway

**Figure 3:**
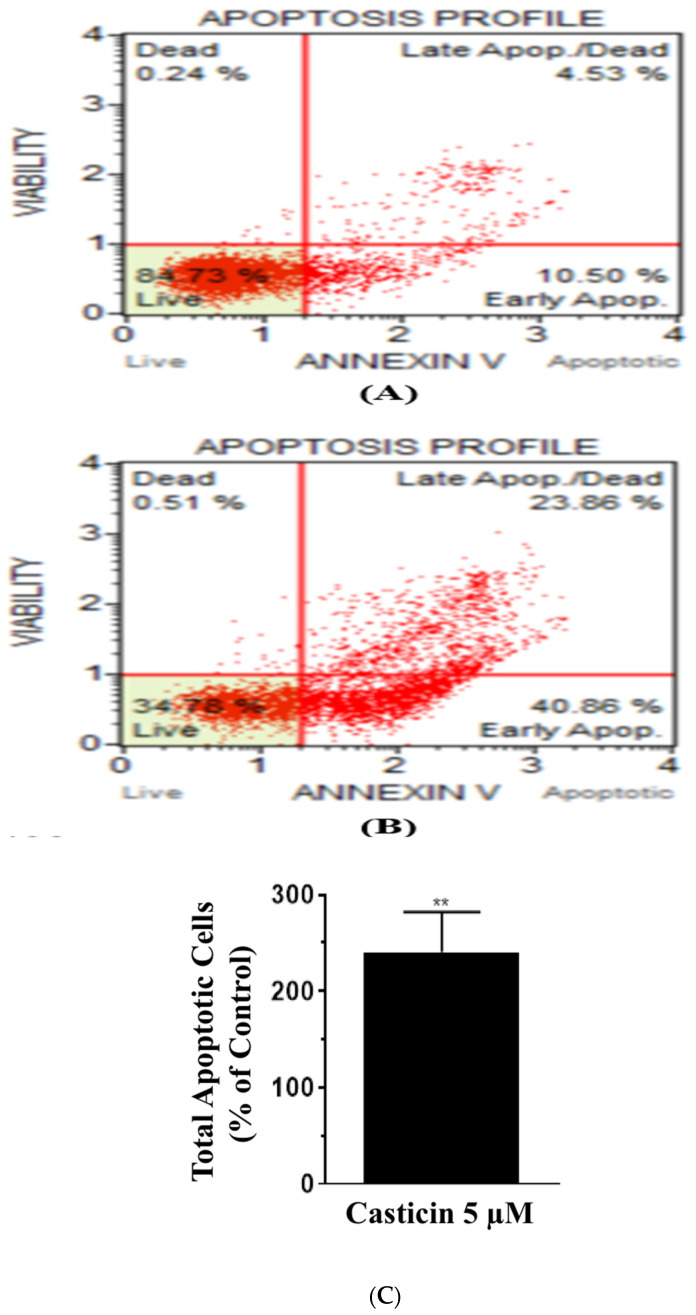
Casticin induces apoptosis in DLD-1 cells. (**A**) Representative annexin scatter plot of the control cells, (**B**) casticin-treated sample. The analysis was done after 48 h of treatment. Total apoptotic cells normalized to the control with the means and standard deviations from 7 trials are shown in bar graph (**C**). Total number of apoptotic cells increased significantly in casticin-treated group. Two-tailed unpaired *t*-test, alpha set to 0.05, significant change in *p* value < 0.0044 (**).

To investigate the mode of action of casticin, Annexin V Muse flow cytometric assay was performed on DLD-1 cells at the IC_50_ concentration of 5 µΜ and on incubation for 48 h. Representative scatter plot of the control sample is shown in Figure 3A and treatment with casticin (Figure 3B). An increase in total apoptotic cells in casticin-treated samples when compared to the control samples was observed from the data in the scatter plots. Total apoptotic cells as percent of control is represented in (Figure 3C). Further analysis of programmed cell death revealed that multicaspaes were induced by casticin treatment (Supplementary Materials, Figure S1).

### 3.4. Casticin Impacts Bcl-2 Levels in DLD-1 Cells

**Figure 4:**
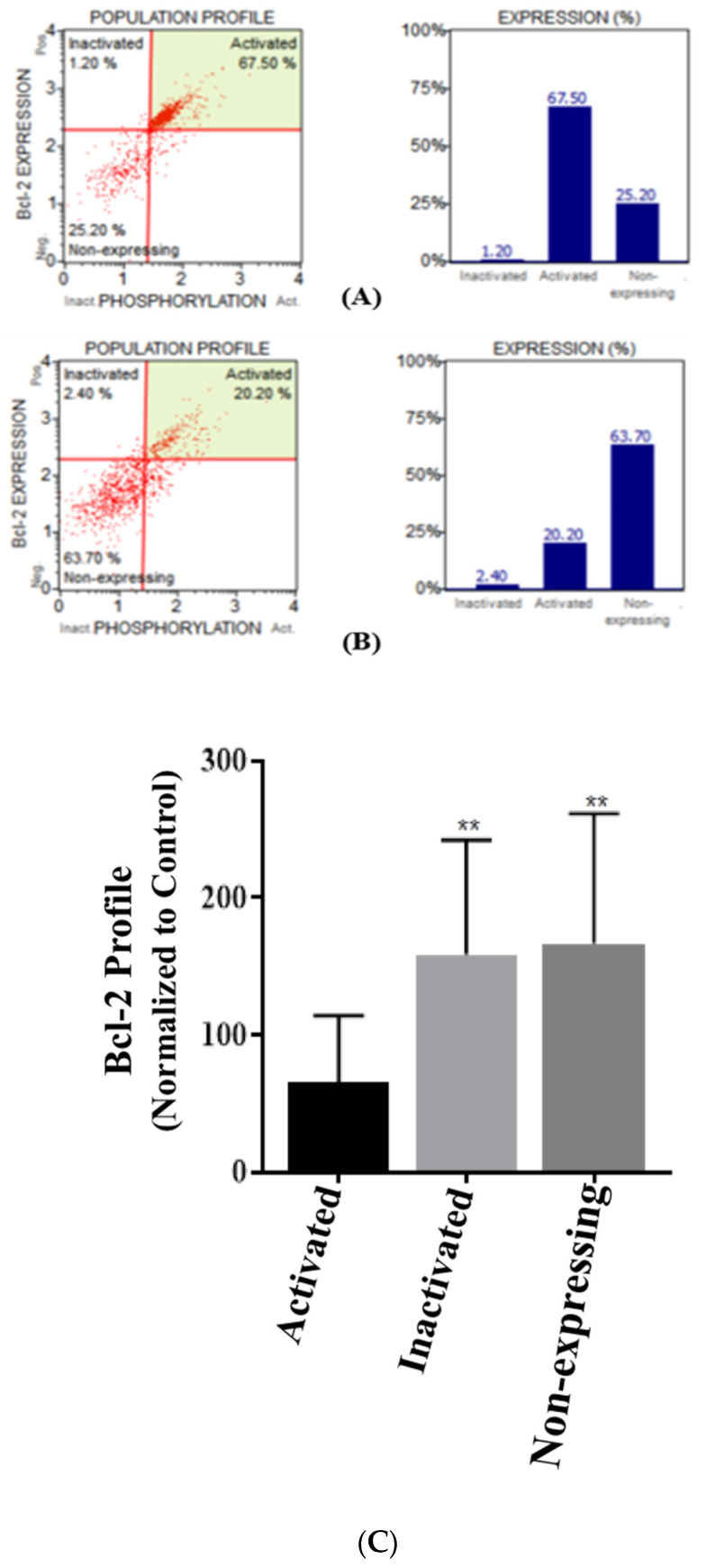
Expression profile of Bcl-2 levels on treatment with casticin in DLD-1 cells. (**A**) Representative Bcl-2 scatter plot of DLD-1 control cells. (**B**) Casticin-treated sample. (**C**) On normalizing to the control group, the activated population, inactivated population, and non-expressing cells in the treatment group are represented. Two-way ANOVA with Sidak’s multiple comparison, alpha set to 0.05, and *p* value for inactivated and non-expressing cells is 0.008(**)8 and 0.0035 (**), respectively.

Bcl-2 plays a pivotal role in cell survival/cell death signaling pathway in cancers. We opted to investigate the effect of casticin on this key moiety in DLD-1 cells. Representative scatter plot of the Bcl-2 levels of the control group is shown in Figure 4A, casticin (IC_50_ concentration of 5 µM)-treated group (Figure 4B). On normalizing to the control sample as shown in the bar graph (Figure 4C), there was increase in the inactivated and non-expressing cells in the DLD-1 cells treated with casticin. The increase was statistically significant with *p*-values of 0.0088 for the inactivated population and 0.0035 for the non-expressing cells. 

### 3.5. Casticin Induces Cell Cycle Arrest in DLD-1 Cells

**Figure 5:**
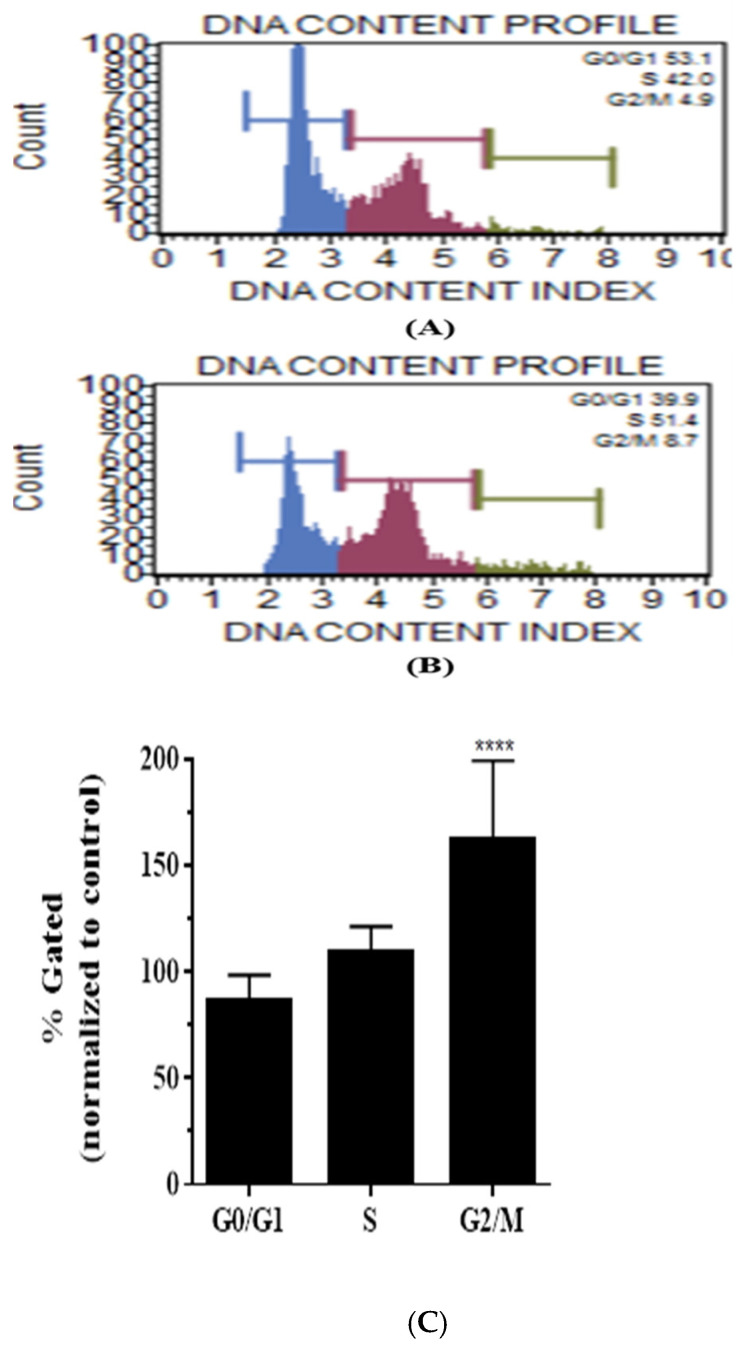
Cell cycle profiles of control group and casticin-treated group in DLD-1 cells. Representative cell cycle Muse scatter plots of DLD-1 control cells (**A**), casticin (5 µM)-treated sample (**B**). On normalizing to the control sample, a statistically significant increase in cells in the G2/M phase of the cell cycle was observed. Means with standard deviations are represented by bar graphs (**C**). Two-way ANOVA with Sidak’s multiple comparison, Alpha set to 0.05, significant increase in G2/M population, and a *p* value < 0.0001 (****) is represented.

To further delineate the impact of casticin on DLD-1 cells, its effect on the cell cycle was investigated by performing the cell cycle Muse assay. The different phases of the cell cycle in the control sample are shown in Figure 5A, phases of the cell cycle in cells treated with 5 μM of casticin (Figure 5B). Casticin treated DLD-1 cells showed a significant increase in cells in the G2/M phase with a *p* value < 0.0001 when compared to the control cells, as shown in (Figure 5C). 

## 4. Discussion

In the present study, casticin a specific flavonoid (tetramethoxyflavone) was tested for its potential to inhibit the viability of the colorectal adenocarcinoma-DLD-1 and Caco-2 cells and the colorectal carcinoma HCT116 cells. Casticin has been reported to induce either apoptosis or cell cycle arrest in breast, gallbladder, liver and other colorectal cancer cells [9,10,11,12,13,14,15]. No studies have been reported on DLD-1 cells; thus, we opted to investigate the effect of casticin on these cells. In the present study, casticin exhibited a dose dependent and potent inhibition of viability at a low concentration of 5 µM. Casticin also inhibited proliferation of DLD-1 cells when assessed by clonogenic assay which revealed that the proliferation potential of DLD-1 cells was affected. Elucidation of the mode of action of casticin revealed its impact on the extrinsic programmed cell death pathway leading to an increase in apoptotic cells. In the colorectal cancer cell line HCT116, induction of apoptosis by casticin has been reported [20]. Notably, casticin showed an impact on the Bcl-2 levels, a key component of cell survival leading to increase in inactivated and non-expressing molecules. Furthermore, treatment with casticin resulted in a significant increase in cells in the G2/M phase of the cell cycle. Moreover, other flavonoids have been reported to arrest cancer cells in the G2/M phase of the cell cycle [21].

Importantly, casticin exhibited multifaceted action including reducing the colony forming efficiency of DLD-1 cells with sustained response. Furthermore, heterogeneous population of cells in the DLD-1 cell line have been reported and is known to cause resistance to the drug camptothecin [22]; thus, for efficacious treatment, a combination of compounds is essential. Natural compounds such as flavonoids have been reported to cause apoptosis and cell cycle arrest in cancer cells, while they have no effect on healthy normal cells [8]. Therefore, compounds such as casticin could be beneficial to combat this aggressive malignancy. Importantly, in our studies with casticin, we observed a significant impact on pivotal signaling pathways such as the inhibition of cell proliferation with sustained response, invoking the apoptosis pathway, significant impact on the key cell survival molecule-Bcl-2, and significant arrest of cells in the G2/M phase of the cell cycle. Therefore, in vivo studies are warranted, which could result in better treatment for colorectal cancers, with the possibility of less side effects with natural compounds such as casticin or in combination with 5-FU-the first line therapy currently in the clinic for colorectal adenocarcinomas to reduce resistance and increase efficacy.

## Data Availability

All the data relating to this article is presented in the manuscript.

## Supplementary Materials

The following supporting information can be downloaded at: www.mdpi.com/article/10.3390/genes13050815/s1, Figure S1: Multicaspases elicited during induction of apoptosis by casticin.
