# Supplementary material for: Casticin Impacts Key Signaling Pathways in Colorectal Cancer Cells Leading to Cell Death with Therapeutic Implications
Source: Genes (Basel). 2022 May 3;13(5):815. doi: 10.3390/genes13050815 (PMC9141418; doi:10.3390/genes13050815)
Supplement: Supplementary file 1 [file genes-13-00815-s001.zip › Supplementary Figure S1.pdf]

Supplementary Figure S1

Multicaspase was elicited during induction of apoptosis by casticin.
In an attempt to further elucidate the pathway of apoptosis observed in casticin treated DLD-1 cells, a multicaspase Muse assay was performed on treating DLD-1 cells with casticin at the IC_{50} concentration of 5 μM and on incubation for 48 hours. Representative scatter plot of the control sample is shown in Supp. Fig. 1(**A**)**,** treatment with casticin (**B**). An increase in total multicaspase positive cells in casticin treated sample when compared to the control sample was observed from the scatter plots. Total multicaspase positive cells as percent of control is shown by bar graph in (**C**)**.** On normalizing to the control after 5 independent trials, there was statistically significant increase in multicaspase activation.

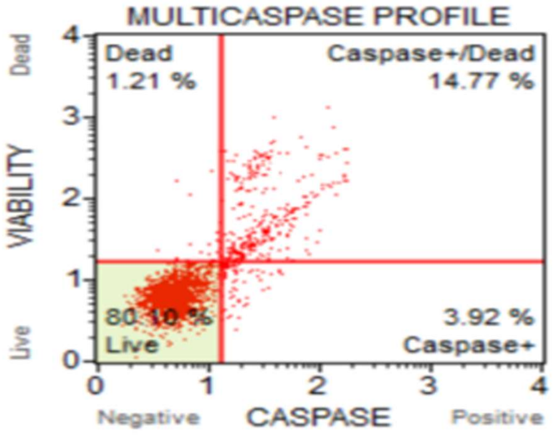

(A)

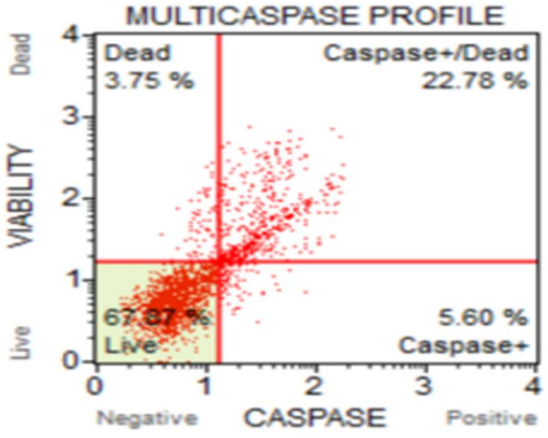

(B)

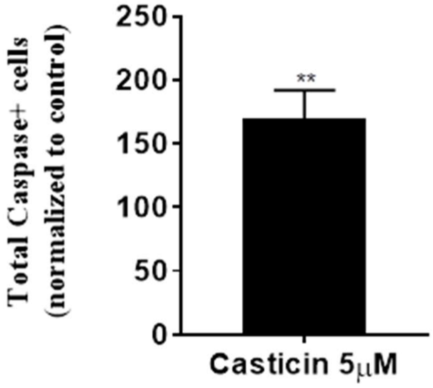

(C)

Supplementary Figure S1. Casticin has impact on multicaspase in DLD-1 cells. Representative scatter plot of the control cells **(A)**, casticin (5 μM) treated sample (**B)**. Cells were treated for 48 hours with the compound. On normalizing to the control, the increase in multicaspase in each of the treatment group is represented by bar graph (**C**). Two-tailed unpaired t-test, alpha set to 0.05 was performed, p value < 0.0014.
